# Assessing adverse childhood experiences, social, emotional, and behavioral symptoms, and subjective health complaints among Hungarian adolescents

**DOI:** 10.1186/s13034-021-00365-7

**Published:** 2021-02-22

**Authors:** Beáta Kovács-Tóth, Barnabás Oláh, Gábor Papp, Ildikó Kuritárné Szabó

**Affiliations:** 1 Department of Behavioural Sciences, University of Debrecen Faculty of Medicine, Nagyerdei krt. 98, POB. 45, Debrecen, 4032 Hungary; 2 Doctoral School of Health Sciences, University of Debrecen, Debrecen, Hungary; 3 Institute of Psychology, University of Debrecen, Debrecen, Hungary

**Keywords:** Adolescence, Adverse childhood experiences, Emotional symptoms, Behavioural symptoms, Health complaints

## Abstract

**Background:**

Adverse Childhood Experiences (ACEs) can have lifelong adverse impacts; they can play a role in the development of subsequent emotional, cognitive, and social impairments leading to somatic and mental difficulties, as well as health damaging behaviours. Unfortunately, there are currently no research data available in Hungary regarding the frequency of ACEs among adolescents.

**Aims:**

A cross sectional questionnaire survey was conducted in a community sample of Hungarian adolescents to assess the frequency of ACEs and analyse their association with current social, emotional, and behavioural symptoms (SEB), and subjective health complaints (SHC).

**Methods:**

Demographic data, ACEs, SEB and SHC status of 516 adolescents aged 12 to 17 were collected. ACEs were assessed using the ACE Score Calculator; for SEB the Strengths and Difficulties Questionnaire, and for SHC some specific items from the Health Behaviour of School Children questionnaire were employed. To analyse the relationship of ACEs to SEB and SHC logistic regression was performed.

**Results:**

Our results showed that the frequency of ACEs, SEB and SHC is high among adolescents. One-fourth of the students reported ≥ 2 categories of childhood exposures, and 7.4% reported having experienced ≥ 4 types of ACEs. The most prevalent forms of child maltreatment were emotional neglect (15.5%) and emotional abuse (14.5%). The most frequent dysfunctional household condition was parental divorce or separation (23.8%), followed by household substance abuse (8.9%) and household mental illness (8.1%). Almost one-fifth of students (17.5%) reported SEB symptoms (peer relationship problems in 21.7%, emotional symptoms in 14.6%, conduct problems in 18.3%, hyperactivity in 15%). The prevalence of SHC was also high: more than half of the students experienced at least one subjective health complaint multiple times a week. Significant associations were found between ACEs and the SEB/SHC reported by students.

**Conclusions:**

Adverse childhood experiences, social, emotional, and behavioural symptoms, and SHC are common among Hungarian adolescents. The cumulation of ACEs is associated with a higher number of SEB and SHC symptoms. Therefore, prevention programmes, early recognition, risk reduction, and therapy are needed.

## Background

Adverse childhood experiences (ACEs) are traumatic childhood events occurring within one’s family (intrafamilial) before the age of 18, which can include physical, sexual and emotional abuse, emotional and physical neglect, and dysfunctional household conditions (separation/divorce, witnessing violent treatment of mother, household substance abuse, household mental illness, incarcerated household member) [1]. As studies show, the prevalence of child maltreatment and household dysfunctions is high worldwide: almost half of adult individuals experienced at least one ACE, which indicates this is a serious public health issue [2–4].

The frequency of ACEs among adolescents has also been examined in a few studies based on self-report; the number of these studies is still lower compared to the studies conducted in on adult populations, though [5–8]. Moreover, there is only a limited amount of research data available on children and adolescents from Central and Eastern European countries [9–12].

### Consequences of adverse childhood experiences

Adverse childhood experiences can be considered important pathogenic factors that affect overall personality development and cause substantial adverse health consequences through the whole lifespan. The exposure to a higher number of ACEs is associated with a higher risk for health outcomes [13, 14]. Data in literature have demonstrated a clear relationship between ACEs and a variety of pathogenic health behaviours and outcomes in adulthood including alcoholism [15], drug abuse [16], sexual risk behaviours [17], depression [18], suicide [19], obesity [20], heart disease [21], cancer [22], among others. Furthermore, adversity exposure in childhood increases the risk for perpetration and revictimization throughout the lifetime [23, 24].

Studies on younger people have examined various aspects of exposure to ACEs. Some have demonstrated that exposure to ACEs is related to health complaints in younger children [25]. Another study has shown that early physical maltreatment predicts psychological and behavioural symptoms in adolescents [26]. Besides, there are a few studies that have found a relationship between ACEs and depression [27], anxiety, obesity [28], bronchial asthma [29], the risk for perpetration and revictimization [30], school success [31], substance use [32] in adolescents. Abused adolescents demonstrate more risky behaviours, e.g. having unprotected sex [33]. Low self-esteem and predisposition to aggression may also be serious psychological sequelae of child maltreatment and exposure to parental violence. Furthermore, correlation has also been found between cumulative exposure to adversity and poor self-rated health in adolescence [34].

The aim of this study is to assess—for the first time in Hungary (a Central Eastern European country/post-communist country)—the frequency and types of ACEs, and a wide range of symptoms, namely social, emotional and behavioural symptoms (SEB), and subjective health complaints (SHC) occurring among adolescents. Next, we also aimed to examine the association of ACEs with this broad spectrum ranging from social, emotional and behavioural symptoms (SEB) to subjective health complaints (SHC).

## Methods

### Sampling and data collection

Data collection was conducted between March 2018 and January 2020 in schools from four counties that responded to our request. We contacted 12 schools from 7 settlements and picked the schools in line with the type of the settlement in order that both village schools and a variety of town or city schools be represented in the sample. We visited grade 7 to 10 students whose age ranged from 12 to 17 years. Altogether 516 adolescents were included. (The relevant CONSORT flow diagram is provided among the Additional file 1: Figure S1). After their parents signed the parental informed consent, the children participating in the study also gave their written informed consent to take part in the study. Data collection was performed in groups, under the assistance of Health psychology students, where needed, in the framework of a class master’s class. Students filled in the questionnaires anonymously.

Ethics approval was issued by the Research Ethics Committee of the Hungarian Medical Research Council (Egészségügyi Tudományos Tanács) under the approval number ETT TUKEB 47848-7/2018/EKU.

The demographic features of the sample are provided in Table 1.

**Table 1:** Demographic characteristics of the sample

Sociodemographic variable	Boys (N = 208)	Girls (N = 308)	Total (N = 516)
Age mean (SD)	15.28 (1.04)	15.28 (1.14)	15.28 (1.10)
Location N (%)			
Village	70 (33.7)	81 (26.2)	151 (29.2)
Town	114 (54.8)	154 (50.2)	268 (52.0)
City	24 (11.5)	73 (23.6)	97 (18.8)
Maternal education N (%)			
Primary or less than primary	29 (16.2)	39 (13.9)	68 (14.8)
Secondary	78 (43.6)	128 (45.6)	206 (44.8)
Tertiary	72 (40.2)	113 (40.6)	185 (40.4)

### Measures

The data were obtained with the help of a self-report questionnaire battery. Besides demographic data (gender, age, location, maternal education), the ACE Score Calculator, the Strengths and Difficulties Questionnaire (SDQ), and the items relating to subjective health complaints of the HBSC Questionnaire were employed.

### Adverse childhood experiences

The ACE Score Calculator is a self-report retrospective questionnaire containing 10 items [35, 36]. It assesses the exposure to 10 types of intrafamilial ACEs, including 5 types of abuse (emotional, physical, sexual abuse, physical and emotional neglect), and 5 types of dysfunctional family environment (parental separation/divorce, witnessing violent treatment of mother, household substance abuse, household mental illness, incarcerated household member) by asking 10 questions to which a yes/no answer must be given. In order to reduce subjectivity of perception, the survey asks questions about concrete behaviour patterns by giving examples. On the basis of the types of adverse childhood experiences the person underwent a cumulative ACE score is calculated, which is an integer number between 0 and 10. The cumulative ACE score is a severity index emphasizing the accumulation of the types of experiences, which indicates how many types of adversities a person has experienced in his/her childhood. The English version of the ACE Score Calculator was translated into Hungarian by the present authors; and cross-cultural adaptation was carried out using an iterative forward–backward translation sequence relying on an independent native speaker. Item contents and item response options are provided among the (Additional file 1: Table S1).

### Strengths and Difficulties Questionnaire (SDQ)

The Strengths and Difficulties Questionnaire (SDQ) was developed to assess the behavioural characteristics of 4- to 16-year-old children [37, 38]. (The informant-rated version of the SDQ can be completed by either the parents or teachers of the involved children). In this study the self-reported version was applied. The 25 items of the questionnaire can be grouped in 5 factors: hyperactivity, emotional symptoms, conduct problems, peer relationship problems and prosocial. Response categories were: not true (0), somewhat true (1), certainly true (2). First, a total needs to be calculated for each subscale (up to 10 points); then by summing the totals of the subscales of peer relationship problems, emotional symptoms, conduct problems, and hyperactivity we can work out the total difficulties score (up to 40 points). Based on totals and the defined cut-off point individuals can be grouped into three categories: normal, borderline, and abnormal. Higher scores are indicative of a higher number of problems in adolescents. Emotional symptoms are assessed with the help of items that read “I worry a lot”, or “I am often unhappy, depressed or tearful”. Among the items assessing behavioural symptoms there are statements like “I am often accused of lying or cheating”. The item that reads “I get very angry and often lose my temper” will mostly detect conduct and behavioural problems. One of the items referring to peer relationship problems is for example: “I have one good friend or more” and “Other children or young people pick on me or bully me”. Hyperactivity is assessed on the basis of items like “I am restless, I cannot stay still for long” or “I am constantly fidgeting or squirming”. The Hun-garian version of the Strengths and Difficulties Questionnaire was adapted and validated by Birkás et al. [39].

As adolescents with subthreshold scores may also experience several difficulties in everyday functioning—which may divert the developmental pathway (in this critical age group) into an unfavourable direction—we treated the borderline and abnormal categories in combination when reporting the frequency of SEB symptoms.

### Health Behaviour of School Children (HBSC)

Data were collected using relevant items from the Hungarian questionnaire [40] of the Health Behaviour of School Children (HBSC) study of 2013/2014 [41]. The HBSC is a comprehensive questionnaire for the assessment of school children’s health behaviour, which is conducted every 4 years in more than 40 countries in international cooperation with the World Health Organization, using an agreed methodology [42]. We did not make use of the whole questionnaire but only employed the questions related to subjective health complaints. Subjective health complaints (SHC) include bodily symptoms ranging from temporary bad feelings to chronic clinical conditions requiring permanent medical monitoring, which also impair a person’s everyday functioning. The term “subjective” highlights the function of personal experience and interpretation: it is subjectivity that determines how and to what extent a specific complaint will affect a person’s quality of life. We asked the students about how frequently they experienced nine subjective health complaints (psychological and somatic symptoms): headache, stomachache, backache, feeling low, irritability, nervousness, sleeping difficulties, dizziness, fatigue. (In all categories the answers to choose from were almost every day/multiple times a week/about once a week/about once a month/rarely or never.)

### Statistical analyses

Statistical analyses were performed using IBM SPSS Statistics v. 23. First, the accumulation and prevalence of ACEs, the prevalence of SEB and SHC were described in the sample, overall and by gender. Next, the association of ACE with SEB was assessed using generalized linear models; then the correlation between ACEs and SHC was analysed using logistic regression with entry method. Both analyses were adjusted for age, gender, location and maternal education. As a next step, the frequency categories of SHC were combined in order to reduce the number of categories. Finally, post-test analysis was performed using the adjusted Wald test.

## Results

### Distribution of ACE score

To study the accumulation of adversities we grouped cumulative scores into the 5 categories Felitti et al. [1] also used, namely 0, 1, 2, 3, 4 or more ACEs reported. In our Hungarian adolescent population, 51.9% (n = 264) reported that they had not experienced any childhood adversity. Almost one-fourth, 22.1% (n = 114) of the sample reported one childhood adversity, 10.3% (n = 53) reported two types of ACEs, and 6.0% (n = 31) reported three ACEs. Four or more ACEs had been experienced by 7.4% (n = 38) of respondents (Pearson’s Chi squared test: p = 0.008). One-fourth (23.7%) reported multiplex, meaning more than 2 categories of childhood exposures.

As regards gender, the Pearson’s Chi-squared test indicated that the accumulation of adversities is significantly more frequent among girls: 10.1% reported 4 or more adversities, and 29.2% reported two or more adverse experiences. Moreover, almost twice as many girls as boys reported 3 adversities, and two and a half times more girls had experienced ≥ 4 adversities compared to boys.

The distribution by gender of ACE accumulation is shown in Table 2.

**Table 2:** Distribution of ACE score overall and by gender

ACE score	Boys N = 202 N (%)	Girls N = 298 N (%)	Total N = 500 N (%)	p-value
0	113 (55.9)	151 (50.7)	264 (51.9)	0.017*
1	54 (26.7)	60 (20.1)	114 (22.1)	
2	19 (9.4)	34 (11.4)	53 (10.3)	
3	8 (4.0)	23 (7.7)	31 (6.0)	
≥ 4	8 (4.0)	30 (10.1)	38 (7.4)	

*Pearson’s Chi-squared test

### The prevalence of adverse childhood experiences

The most prevalent form of reported child maltreatment was emotional neglect (15.5%, n = 80), and emotional abuse (14.5%, n = 75). The least prevalent reported child maltreatment was psychical neglect (3.9%, n = 20).

The most frequent reported dysfunctional household condition was parental divorce or separation (23.8%, n = 123), followed by household substance abuse (8.9%, n = 46) and household mental illness (8.1%, n = 42). The least prevalent reported household dysfunction was having witnessed violent treatment of mother (4.1%, n = 21).

Among girls, emotional neglect (21.4%, n = 65) and emotional abuse (18.1%, n = 55) were the most prevalent reported maltreatments. Among boys, emotional abuse (9.7%, n = 20) and emotional neglect (7.3%, n = 15) were the most frequent.

As regards the reported prevalence of adverse childhood experiences by genders, we found a significant difference in three cases. Emotional abuse, emotional neglect, and household mental illness were more prevalent in girls. Although the difference cannot be considered significant, girls reported double prevalence of witnessing violent treatment of mother compared to boys, and household substance abuse was reported by nearly three times as many girls as boys. The prevalence of adverse childhood experiences in the sample, overall and by the gender is shown in Table 3.

**Table 3:** Prevalence of adverse childhood experiences in the sample, overall and by gender

Adverse childhood experiences	Boys N (%)	Girls N (%)	Total N (%)	p-value
Maltreatment				
Emotional abuse (N = 511)	20 (9.7)	55 (18.1)	75 (14.5)	0.008*
Physical abuse (N = 511)	11 (5.3)	22 (7.2)	33 (6.4)	0.385
Sexual abuse (N = 509)	8 (3.9)	18 (5.9)	26 (5.0)	0.310
Emotional neglect (N = 510)	15 (7.3)	65 (21.4)	80 (15.5)	< 0.001*
Physical neglect (N = 509)	8 (3.9)	12 (4.0)	20 (3.9)	0.965
Family dysfunction				
Parental separation/divorce (N = 508)	49 (23.8)	74 (24.5)	123 (23.8)	0.853
Witnessing violent treatment of mother (N = 510)	6 (2.9)	15 (5.0)	21 (4.1)	0.252
Household substance abuse (N = 511)	13 (6.3)	33 (10.9)	46 (8.9)	0.076
Household mental illness (N = 507)	10 (4.9)	32 (10.6)	42 (8.1)	0.023*
Incarcerated household member (N = 506)	17 (8.3)	23 (7.6)	40 (7.7)	0.790

*Pearson’s Chi-squared test

### The prevalence of social, emotional, and behavioural symptoms

Table 4 indicates the mean scores and the standard deviation of the individual SDQ scales. The value of the Cronbach-α (a reliability index) was 0.715 in this sample.

**Table 4:** Social, emotional, and behavioural (SEB) mean scores in the sample, overall and by gender

SEB symptoms	Boys mean (SD)	Girls mean (SD)	Total mean (SD)
Emotional symptoms (N = 507)	2.13 (2.14)	2.91 (2.52)	2.6 (2.41)
Conduct problems (N = 508)	2.57 (1.73)	2.25 (1.38)	2.37 (1.54)
Hyperactivity/inattention problems (N = 506)	3.59 (1.92)	3.83 (1.81)	3.73 (1.86)
Peer relationship problems (N = 504)	2.35 (1.73)	2.43 (1.52)	2.4 (1.60)
Total difficulties (N = 503)	10.45 (5.3)	11.39 (4.92)	11.0 (5.09)

The comparison of the prevalence of SEB symptoms between individuals belonging to the normal, borderline, or abnormal category, as well as the comparison of prevalence between genders is provided in Table 5. Borderline category means that “This score is slightly raised, which may reflect clinically significant problems” [43].

**Table 5:** Prevalence of social, emotional, and behavioural (SEB) symptoms, overall and by gender

SEB symptoms	N (%)				p-value
	Gender	Normal	Borderline	Abnormal	
Emotional symptoms (N = 507)	Boys	183 (90.6)	10 (5.0)	9 (4.5)	0.018*
	Girls	250 (82.0)	22 (7.2)	33 (10.8)	
	Total	433 (85.4)	32 (6.3)	42 (8.3)	
Conduct problems (N = 508)	Boys	150 (74.3)	22 (10.9)	30 (14.9)	0.001*
	Girls	265 (86.6)	21 (6.9)	20 (6.5)	
	Total	415 (81.7)	43 (8.5)	50 (9.8)	
Hyperactivity/inattention problems (N = 506)	Boys	165 (82.5)	17 (8.5)	18 (9.0)	0.194
	Girls	265 (86.6)	14 (4.6)	27 (8.8)	
	Total	430 (85.0)	31 (6.1)	45 (8.9)	
Peer relationship problems (N = 504)	Boys	156 (78.0)	28 (14.0)	16 (8.0)	0.022*
	Girls	239 (78.6)	56 (18.4)	9 (3.0)	
	Total	395 (78.4)	84 (16.7)	25 (5.0)	
Total difficulties (N = 503)	Boys	164 (82.4)	20 (10.1)	15 (7.5)	0.991
	Girls	251 (82.6)	31 (10.2)	22 (7.2)	
	Total	415 (82.5)	51 (10.1)	37 (7.4)	

*Pearson’s Chi-squared test

The prevalence of symptoms was as follows: emotional symptoms (8.3%, n = 42), conduct problems (9.8%, n = 50), hyperactivity/inattention problems (8.9%, n = 45), peer relationship problems (5%, n = 25), total difficulties (7.4%, n = 37).

The prevalence of possibly occurring clinically significant problems is indicated by the sum of the numbers in the borderline and abnormal categories: emotional symptoms 14.6% (n = 74), conduct problems 18.3% (n = 93), hyperactivity/inattention problems 15.0% (n = 76), and peer problems 21.7% (n = 109). Regarding gender, boys reported significantly more conduct problems (24.9%, n = 52), whereas girls reported a significantly higher prevalence in peer relationship problems (21.4%, n = 65) and emotional problems (18%, n = 41).

### The prevalence of subjective health complaints

Table 6 presents the prevalence of symptoms related to subjective health complaints occurring daily or at least multiple times a week both overall and by gender.

**Table 6:** Prevalence of subjective health complaints (SHC) overall and by gender

SHC (multiple times a week)	Boys N (%)	Girls N (%)	Total N (%)	p-value
Headache (N = 514)	31 (15.0)	103 (33.6)	134 (26.1)	< 0.001*
Stomachache (N = 514)	22 (10.6)	63 (20.5)	85 (16.5)	0.003*
Backache (N = 514)	25 (12.1)	67 (21.8)	92 (17.8)	0.005*
Feeling low (N = 513)	38 (18.4)	110 (35.9)	148 (28.6)	< 0.001*
Irritability (N = 513)	32 (15.5)	74 (24.2)	106 (20.5)	0.017*
Nervousness (N = 512)	64 (31.1)	130 (42.5)	194 (37.5)	0.009*
Sleeping difficulties (N = 510)	44 (21.3)	84 (27.7)	128 (24.8)	0.098
Dizziness (N = 513)	17 (8.3)	52 (16.9)	69 (13.3)	0.005*
Fatigue (N = 512)	90 (43.7)	188 (61.4)	278 (54.8)	< 0.001*

*Pearson’s Chi-squared test

Over half of the adolescents (54.8%, n = 278) reported fatigue, more than one-third (37.5%, n = 194) revealed nervousness and (28.6%, n = 148) feeling low; one-quarter (26.1%, n = 134) said they suffered from headache multiple times a week and had difficulty falling asleep (24.8%, n = 128). The rest of the subjective health complaints were also reported in the studied sample. Except for sleeping difficulties, all the measured SHC were significantly more frequent in girls than boys.

### Associations between the number of adverse childhood experiences (ACE) and social, emotional, and behavioural (SEB) problems

We used generalized linear models of ACE with SEB adjusted for age, gender, location and maternal education. Table 7 shows that adolescents with two, three, and four or more ACEs reported more overall difficulties in comparison with adolescents with no ACEs. This association was strong and cumulative. When separately modelling the association of ACE with social, emotional and behavioural symptoms, a similar association was found with lower B coefficients.

**Table 7:** Associations between the number of adverse childhood experiences (ACEs) and social, emotional, and behavioural (SEB) symptoms, separately and overall

	Emotional symptoms		Conduct problems		Hyperactivity/inattention problems		Peer problems		Total difficulties	
	B	p-value	B	p-value	B	p-value	B	p-value	B	p-value
ACE score										
0	Ref.		Ref.		Ref.		Ref.		Ref.	
1	− 0.030	0.912	0.166	0.316	0.027	0.899	− 0.196	0.273	− 0.110	0.842
2	1.076	0.002*	0.691	0.001*	1.048	< 0.001*	0.318	0.177	3.139	< 0.001*
3	0.761	0.110	0.803	0.005*	1.249	0.001*	0.782	0.011*	3.672	< 0.001*
4 or more	1.461	0.001*	0.922	0.001*	1.077	0.002*	0.779	0.007*	4.282	< 0.001*
Model	χ^2^(10) = 49.172 p < 0.001		χ^2^(10) = 37.068 p < 0.001		χ^2^(10) = 32.298 p < 0.001		χ^2^(10) = 25.895 p = 0.004		χ^2^(10) = 59.623 p < 0.001	

*Generalised linear models, adjusted for age, gender, location, and maternal education

### Associations between the number of adverse childhood experiences and subjective health complaints

Logistic regression with entry method was performed to analyse the association of ACEs with SHC adjusted for age, gender, location and maternal education. After combining the categories of SHC recurrence, we defined frequency as a binary variable (multiple times a week vs. less than multiple times a week). Table 8 shows that adolescents with moderately accumulated ACEs had several times higher odds of experiencing SHC multiple times a week in comparison with adolescents reporting no ACEs.

**Table 8:** Associations between the number of adverse childhood experiences (ACEs) and subjective health complaints (SHC)

	Headache		Stomachache		Backache		Feeling low		Irritability	
	OR	p-value	OR	p-value	OR	p-value	OR	p-value	OR	p-value
ACE score										
0	Ref.		Ref.		Ref.		Ref.		Ref.	
1	1.23	0.506	1.20	0.636	1.90	0.053	1.36	0.297	1.58	0.152
2	2.50	0.010*	2.12	0.074	1.33	0.529	2.47	0.010*	2.07	0.056
3	3.03	0.014*	6.44	< 0.001*	6.56	< 0.001*	3.61	0.004*	2.76	0.030*
4 or more	2.80	0.015*	3.96	0.003*	2.81	0.021*	4.98	< 0.001*	2.87	0.015*
Model	χ^2^(10) = 54.349 p < 0.001		χ^2^(10) = 49.573 p < 0.001		χ^2^(10) = 36.633 p < 0.001		χ^2^(10) = 59.091 p < 0.001		χ^2^(10) = 32.115 p < 0.001	

	Nervousness		Sleeping difficulties		Dizziness		Fatigue	
	OR	p-value	OR	p-value	OR	p-value	OR	p-value
ACE score								
0	Ref.		Ref.		Ref.		Ref.	
1	0.92	0.753	1.40	0.243	1.30	0.499	0.93	0.751
2	2.82	0.002*	1.73	0.129	1.38	0.489	2.49	0.010*
3	6.32	< 0.001*	2.48	0.043*	0.87	0.832	2.93	0.025*
4 or more	1.83	0.140	2.41	0.036*	3.24	0.013*	1.92	0.129
Model	χ^2^(10) = 50.874 p < 0.001		χ^2^(10) = 21.400 p = 0.018		χ^2^(10) = 24.781 p = 0.001		χ^2^(10) = 34.703 p < 0.001	

*Logistic regression with entry method, adjusted for age, gender, location, and maternal education

Exposure to two ACEs increased the odds of reporting headache (OR = 2.5), feeling low (OR = 2.47), nervousness (OR = 2.82), and fatigue (OR = 2.49) multiple times a week compared to no childhood exposure. Reporting three ACEs increased the odds of reporting headache multiple times a week by 3.03 times, of stomachache by 6.44 times, of backache by 6.56 times, of nervousness by 6.32 times, of sleeping difficulties by 2.48, and fatigue by 2.93 times in comparison with adolescents without ACEs. Four or more ACEs also significantly increased the odds of reporting headache (OR = 2.80) and sleeping difficulties (OR = 2.41) to an extent similar to three ACEs; however, in the case of stomachache (OR = 3.96) and backache (OR = 2.81) this increase was much less marked compared to the case with three ACEs. As for nervousness and fatigue, we found no statistically significant relationship between exposure to four or more ACEs and the frequency of these complaints. Adolescents with four or more ACEs had the highest odds and were 4.98 times more likely to report feeling low, 2.87 times more likely to report irritability and 3.24 times more likely to report dizziness multiple times a week in comparison with adolescents without ACE.

## Discussion

### Adverse childhood experiences

This study shows that the frequency of reported child maltreatment and other ACEs is relatively high in this population of Hungarian adolescents: almost half of this sample reported some intrafamilial adversity during their 12 to 17-year-long life, one-fourth of the students reported ≥ 2 categories of childhood adversity exposures, and 7.4% reported experiencing ≥ 4 types of ACEs.

The most prevalent forms of reported child maltreatment were emotional neglect (15.5%) and emotional abuse (14.5%). The most frequently reported dysfunctional household condition was parental divorce or separation (23.8%). The rest of all reported adversities were below 10%. Out of all adversities physical neglect was the least frequent (3.9%), which also shows that only a small proportion of the Hungarian adolescents we studied suffers from not living in appropriate material/physical environment conditions.

As the duration of exposure to ACEs is shorter than 18 years, the data on prevalence must inevitably be lower than in studies conducted in adults.

Every type of ACE (except for incarcerated household member) was more prevalent in girls than boys, and the same was true for cumulative score. To account for this significant difference across genders (emotional neglect is triple, emotional abuse is double in girls than boys), we may assume girls be more sensitive to emotional attitude and related deficiencies, and expect more/warmer interactions. Girls talk more about the emotional aspect of their experiences than boys. In addition, girls use more emotional words when discussing scary events than boys [44]. Girls are also more willing to disclose/express negative emotions such as sadness and fear [45].

In general, girls seem to be more liable to recognise and/or admit experiencing ACEs. Furthermore, systematic links were found between adolescent problem status and parent approaches to emotion socialisation [46]. These claims are also supported by our own clinical experiences. Considering the unexpected and unjustifiable difference between genders in terms of the frequency of household substance abuse and household mental illness, we can again assume that girls are more disposed to report, and this must have prevailed in case of all the adversities.

Our sample was taken from a country where—unlike in the USA—the awareness of the population has not been raised toward this problem; there is no education provided and no research has been done in the topic. Regarding child maltreatment, it is only the area of sexual abuse where research has consistently confirmed a higher proportion of female victims than males [4, 47].

Unfortunately, we have found few comparable data in Central Eastern European adolescent population in relation to ACEs; most research has been conducted in adult samples. Considering the research done in neighboring countries—which are also post-Soviet countries—we have found that two studies in Romanian adolescents described a higher prevalence of ACEs. It must be mentioned, though, that the measure they used differed from ours [9, 10]. A Slovakian study in adolescents published data on the prevalence of ACEs, which are quite similar to the Hungarian data [11]. As for the Czech study, in which university students were included, we again saw similar results regarding reported emotional abuse, sexual abuse and parental divorce; the rest of adversities (with the exception of incarcerated household member) appeared in higher percentages in the sample [12].

Studying the research data (some data were collected again from samples of older individuals) from eight Eastern European Countries (Albania, Latvia, Lithuania, Montenegro, Romania, Russian Federation, The former Yugoslav Republic of Macedonia, Turkey), it becomes clear that in terms of ACEs Hungary is a middle-ranking country [48]. At the same time, our data regarding emotional neglect, emotional abuse and parental divorce are less favorable, which should definitely raise our awareness. These results are summarised in Additional file 1: Table S2—A comparison of prevalence rates of child maltreatment reported by children, adolescents and adults in selected European countries. The table can be found among the Additional file 1: Table S2.

### Social, emotional, and behavioural symptoms

Every sixth adolescent reported emotional symptoms (the questions mostly assessed the presence of certain features of anxiety and depression), and nearly one-fifth of the sample reported conduct problems. Hyperactivity/inattention problems were outlined by 15.0% of the sample. The prevalence rate of subthreshold ADHD according to international studies is wide-ranging (0.8–23.1%), the comorbidity of subthreshold ADHD is high, and there are several areas where subthreshold ADHD has a meaningful impact on functioning [49]. Nearly one-fifth of adolescents had peer relationship problems. The presence of friends and peer relationships is a matter of cardinal importance in adolescence; their absence or dysfunction might later lead to severe consequences.

Regarding gender differences, behavioural symptoms are significantly more common in boys, whereas peer relationship and emotional problems are more frequent in girls, as expected. Our results on gender differences are consistent with previous study results [38, 50].

In order to test the comparability of our results, we searched the literature for data in other European countries regarding the prevalence rates of self-reported SEB symptoms based on SDQ. We found detailed data in Austria and Poland, and Czech, French, Greek, Dutch, Spanish, Swiss and British data were also available regarding total difficulties [51, 52]. Concerning total difficulties, our present Hungarian sample is a middle-ranking one among the 10 studied European countries.

### Subjective health complaints

Over half of students experienced at least one subjective health complaint multiple times a week. More than 50% reported fatigue, and over one-third reported nervousness. Three out of 10 students complained of feeling low, and over a quarter of them experienced headaches and suffered from sleeping difficulties. Other subjective health complaints also appear in the examined sample.

Apart from sleeping difficulties, all the assessed SHC was significantly more frequent among girls than boys, as could be expected. This may be due to the fact that girls are more likely to report, or because the tendency to somatize is more common among women in several cultures [53].

Results show that a significant percentage of the adolescents we examined struggles with several SHC, as well as social, emotional, and behavioural symptoms.

We also examined what role the directly and indirectly experienced intrafamilial adversities play in the prevalence of symptoms.

### Association between adverse childhood experiences and social, emotional, behavioural symptoms, and subjective health complaints

Our findings suggest that adverse childhood experiences have a significant impact on adolescents’ mental health status and subjective health. The results show a dose–response relationship between ACEs and SEB where the multiple accumulation of ACEs is associated with more SEB. Multiple accumulation of ACEs also shows a strong dose–response relationship with the frequency of mood-related complaints, with more ACEs having a more pronounced impact. Similarly, the impact of ACEs on the frequency of headache, irritability and sleeping difficulties complaints has also been found to be strong and nearly cumulative. Frequent dizziness complaints were strongly associated with ACEs; however, it was only true when ACEs were severely accumulated (four or more ACEs). Having three ACEs was a very strong predictor of the frequency of stomachache, backache and nervousness, and could also strongly predict the frequency of fatigue complaints. Nevertheless, these relationships are not that graded when ACE is increased to four or more; there the association become less pronounced. Similar data have been published on associations between adverse childhood experiences and social, emotional, behavioural symptoms, and subjective health complaints [10, 11].

Furthermore, a number of longitudinal studies are available on correlations between child maltreatment and adolescent development or subsequent psychiatric morbity in adulthood [54–56].

### Final summary

The prevalence of ACEs, emotional, conduct, and peer relationship problems, and subjective health complaints indicates that adolescents (in our sample) need to face several (previous and/or current) intrafamilial challenges, and mental and health problems at an age when the developmental challenges of adolescence themselves pose a demanding challenge.

These results clearly suggest that the population should be widely informed about the potential adverse consequences of childhood adversities.

Both the subthreshold and suprathreshold social, emotional, and conduct problems, and subjective health complaints require special interventions aimed to reduce these symptoms.

In line with earlier international research, our study reveals that the accumulation of ACEs is associated with more SEB and SHC symptoms. A further triggering factor may be the fact that adolescents exposed to ACEs will probably be less prepared to meet the challenges posed by school and peer relationships.

The main strength of our study is that it was the first study to examine Hungarian adolescents using a validated and internationally recognized measure for the assessment of ACEs, SEB and SHC.

Our results provide further support for research aimed at disclosing the association between ACEs and SEB or SHC in adolescents. A novel feature of our study is that besides SEB it also assessed SHC, which provides a more comprehensive view on the possible consequences of ACEs.

A further strength of our study lies in the fact that it studies adolescents. Evidence shows that it is necessary and justified to include children and adolescents in such research, and the gained data are suitable for analysis [57, 58]. It is also important to get schools involved in prevalence studies, which might result in more detailed and more reliable data.

Like all studies, our study has limitations. Firstly, the fact that it is a cross-sectional study limits the interpretability of data. Secondly, the assessment of not only ACEs, but also SEB and SHC was based on self-report, which might bias results, especially the ones related to externalizing symptoms. The parent and teacher version of the SDQ questionnaire are also available, and their use would have obviously been useful for the aims of our study. Unfortunately, we had to accept we could not use them as we did not possess the Ethics Committee approval required for students’ identification, which would have been necessary for coupling the questionnaires.

Thirdly, the ACE Score Calculator is a short retrospective 10-item measure, which may result in reported ACEs getting lost or biased. Next, when interpreting SEB symptoms, we also incorporated the borderline category into our interpretation. Finally, the sample cannot be regarded representative even though adolescents from a wide range of social backgrounds were included. This, however, makes our sample suitable to capture the current situation in large sections of our society.

## Conclusions

We were among the first in Central Eastern Europe to present data on the effects that ACEs have on adolescents, as well as their relationship with SEB symptoms and SHC complaints.

Our results provide additional evidence for the associations between ACEs and SEB/SHC; they further emphasise the need for social and public health prevention among adolescents exposed to ACEs.

Together with other European countries, we also consider it important to draw attention to these serious problems in Central Eastern Europe as well, which makes it inevitable to document and support our studies with valid measures and data quantified in a scientifically acceptable way, respectively.

If the characteristic features of adolescents are described in this way, data may be more reliable and preventive measures may be easier to design and adjust to population. Adolescence is a particularly important and one of the most critical stages of development. It is the optimal time of life in terms of prevention, risk reduction and early intervention alike, when prevention and therapy interventions can be implemented in school settings, which can result in more efficient changes in this population at risk.

## Supplementary information

**Additional file 1: Figure S1.** Consort flow diagram. **Table S1.** The ACE Score Calculator—preambles, item contents and response options. **Table S2.** A comparison of prevalence rates of child maltreatment reported by children, adolescents and adults in selected European countries.

## Data Availability

All data generated or analysed during this study are included in this published article and its Additional files.

## Abbreviations

ACEs: Childhood experiences
SEB: Social, emotional and behavioral symptoms
SHC: Subjective health complaints
SDQ: Strengths and Difficulties Questionnaire
HBSC: Health Behavior of School Children
