# Supplementary material for: Assessing adverse childhood experiences, social, emotional, and behavioral symptoms, and subjective health complaints among Hungarian adolescents
Source: Child Adolesc Psychiatry Ment Health. 2021 Feb 22;15:12. doi: 10.1186/s13034-021-00365-7 (PMC7901200; doi:10.1186/s13034-021-00365-7)
Supplement: Supplementary file 1 — Additional file 1: Figure S1. Consort flow diagram. Table S1. The ACE Score Calculator—preambles, item contents and response options. Table S2. A comparison of prevalence rates of child maltreatment reported by children, adolescents and adults in selected European countries. [file 13034_2021_365_MOESM1_ESM.docx]

Figure S1. CONSORT flow diagram of the study

Contacted schools (n=12)

Contacted class groups (n=25)

Responded students (n=516)

Exluded students (n=75)

Parental refusal (n=3)

Absence from school (n=72)

Contacted students (n=591)

Table S1. The ACE Score Calculator—preambles, item contents and response options.

| **Item.** | **Preamble and Content** | **ACE Category** |
| --- | --- | --- |
|  | During your life: |  |
| 1a | Did a parent or other adult in the household often or very often ... Swear at you, insult you, put you down, or humiliate you? or Act in a way that made you afraid that you might be physically hurt? | Emotional abuse |
| 2a | Did a parent or other adult in the household often or very often ... Push, grab, slap, or throw something at you? or Ever hit you so hard that you had marks or were injured? | Physical abuse |
| 3a | Did an adult person at least 5 years older than you ever ... Touch or fondle you or have you touch their body in a sexual way? or Attempt or actually have oral, anal, or vaginal intercourse with you? | Sexual abuse |
| 4a | Did you often or very often feel that ... No one in your family loved you or thought you were important or special? or Your family didn’t look out for each other, feel close to each other, or support each other? | Emotional neglect |
| 5a | Did you often or very often feel that ... You didn’t have enough to eat, had to wear dirty clothes, and had no one to protect you? or Your parents were too drunk or high to take care of you or take you to the doctor if you needed it? | Physical neglect |
| 6a | Were your parents ever separated or divorced? | Parental separation/divorce |
| 7a | Was your mother or stepmother: Often or very often pushed, grabbed, slapped, or had something thrown at her? or Sometimes, often, or very often kicked, bitten, hit with a ﬁst, or hit with something hard? or Ever repeatedly hit for at least a few minutes or threatened with a gun or knife? | Witnessing violent treatment of mother |
| 8a | Did you live with anyone who was a problem drinker or alcoholic or who used street drugs? | Household substance abuse |
| 9a | Was a household member depressed or mentally ill, or did a household member attempt suicide? | Household mental illness |
| 10a | Did a household member go to prison? | Incarcerated household member |
|  | a Dichotomous scales–yes/no |  |

**Table S2**. A comparison of prevalence rates of child maltreatment reported by children, adolescents and adults in selected European countries.

| **Country** | **Sample** | **Tools** | **Emotional abuse**  **%** | **Physical abuse**  **%** | **Sexual abuse**  **%** | **Emotional neglect**  **%** | **Physical neglect**  **%** | **Parental divorce/**  **separation**  **%** | **Witnessing v. treatment of mother**  **%** | **Household substance abuse**  **%** | **Household Mental illness**  **%** | **Incarceration**  **%** |
| --- | --- | --- | --- | --- | --- | --- | --- | --- | --- | --- | --- | --- |
| **Germany**  [Cohrdes and](https://www.ncbi.nlm.nih.gov/pubmed/?term=Cohrdes%20C%5BAuthor%5D&cauthor=true&cauthor_uid=32192828) Mauz, 2020 | N = 3704  age 7-17,  18-31 | Childhood Trauma Questionnaire | 17.9 | 5.8 | 5.4 | 25.7 | 19.9 | 18.3 |  | 13.5 | 13.7 | 3.1 |
| **Czeh Republic**  Velemínsky, 2020 | N = 1760  Universities  age >18 | ACE questionnaire | 20.7 | 17.1 | 6.4 |  | 8 | 23 | 22.1 | 20.2 | 13.4 | 1.4 |
| **Surveys in eight eastern European countries**  Bellis et al. 2014      **Albania** | Universities, Colleges,  Secondary and Vocational schools  N = 10696  age 18-25 | ACE  questionnaire | 26.5 | 41 | 19.1 | 16.3 |  | 6.6 | 30.0 | 22.2 | 7.6 | 3.7 |
| **Latvia** | age 17-25 |  | 7.9 | 16.2 | 7.0 | 8.9 |  | 42.3 | 20.3 | 35.1 | 18.8 | 8.3 |
| **Lithuania** | age 18-39 |  | 4.3 | 12.9 | 3.5 | 10.1 |  | 19.8 | 16.5 | 27.7 | 10.3 | 3.5 |
| **Montenegro** | age 18-50 |  | 4.7 | 19.6 | 3.6 | 7.4 |  | 9.5 | 9.2 | 14.1 | 5.6 | 7.2 |
| **Romania** | age 14-66 |  | 8.9 | 23.4 | 5.8 | 7.6 |  | 16.4 | 6.7 | 24.7 | 13.8 | 2.3 |
| **Russian Federation** | age 13-41 |  | 2.9 | 12.8 | 5.6 | 18.4 |  | 16.8 | 13.1 | 10.5 | 8.2 | 5.5 |
| **The former Yugoslav Republic of Macedonia** | age 17-45 |  | 4.9 | 6.9 | 7.4 | 16.7 |  | 3.8 | 1.4 | 12.6 | 6.8 | 4.2 |
| **Turkey** | age 18-41 |  | 3.7 | 14.6 | 6.9 | 8.7 |  | 5.3 | 17.9 | 9.6 | 9.5 | 8.3 |
| **Hungary**  Kovács-Tóth et al. 2020 (our study) | N = 516  students  age 12-17 | ACE  Score Calculator | 14.5 | 6.4 | 5 | 15.5 | 3.9 | 23.8 | 4.1 | 8.9 | 8.1 | 7.7 |
